# Errors in reconstruction of dichroic X-ray orientation tomography due to polarization rotation of the incident beam

**DOI:** 10.1107/S1600577525011051

**Published:** 2026-01-20

**Authors:** Matthew A. Marcus, Harlan Heilman, Kas Andrle, Jayden Plumb

**Affiliations:** a Advanced Light Source Lawrence Berkeley National Laboratory 1 Cyclotron Road Berkeley CA94720 USA; b Department of Physics and Astronomy Washington State University Pullman WA99164 USA; c Materials Science Division Lawrence Berkeley National Laboratory 1 Cyclotron Road Berkeley CA94720 USA; Australian Synchrotron, Australia

**Keywords:** X-ray linear dichroism, tomography, orientation imaging

## Abstract

Dichroic X-ray tomography is a technique in which the crystal orientation or magnetization of a sample is resolved in three dimensions. The best-known uses of this technique are for observation of magnetic moments via circular dichroism, using left- and right-handed circularly polarized X-ray beams. Another variant uses linear dichroism to resolve the crystal orientation. In both these techniques, it is assumed that the absorption of X-rays along a path inside a material can be computed as a line integral of a local absorption coefficient along the ray path. For linear dichroism, this assumption is inaccurate because the polarization of the beam changes along the propagation direction when the optic axis of the material is not aligned along the polarization. In this work, a finite-element Maxwell solver is used to simulate tomography and reconstructions. The propagation effect can lead to significant errors in the reconstructed orientations. These errors may be mitigated by taking data at additional angles or by operating at energies at which the dichroism is weak. An iterative approach is proposed which may allow accurate reconstruction with fewer data than would otherwise be required.

## Abstract 2

Linear dichroic X-ray tomography is a technique in which the linear dichroism of anisotropic materials is used to image crystal orientation in three dimensions. We show that the reconstruction of tomograms is subject to errors due to the change in polarization on propagation through the material and propose strategies for mitigating these effects.

## 1. Introduction and motivation

For many years, X-rays have been used to make three-dimensional images of objects with quantitative measurement of electron density. The common technique for doing this is tomography, in which projection images are taken with the beam propagating through the sample at a number of incident angles. This allows for the reconstruction of the absorption coefficient at each voxel of a 3D volume.

However, X-ray absorption can be more complicated than a single quantity per voxel. Near an absorption edge, materials with an anisotropic crystal or magnetic structure will demonstrate a linear or circular dichroism, respectively. Thus, by varying the polarization as well as the direction of the incident beam, it is possible to measure a multi-component property at each voxel in a sample. For example, by illuminating the sample with right- and left-handed circularly polarized beams, it is possible to reconstruct a charge (scalar) and magnetic (vector) contrast, thus four components per voxel. In the case of linear dichroism, the absorption of the sample must be described as a symmetric 3×3 tensor, thus a six-component quantity. Recent applications of this idea have yielded 2D (Lo *et al.*, 2021 ▸; Donnelly *et al.*, 2016 ▸) and 3D (Apseros *et al.*, 2024 ▸) information which would be difficult to obtain in any other way. Such information has yielded insights into the mechanical performance of biomaterials [see *e.g.* Gilbert *et al.* (2008 ▸) and Stifler *et al.* (2021 ▸)].

The basic assumptions behind most tomographic reconstruction methods are that the absorption of X-rays along a ray direction may be described as a line integral of an effective absorption coefficient along the ray, and that the absorption coefficient at a point is independent of those at other points. For scalar and magnetic circular dichroic (MCD) tomography, these assumptions can be accurate because the polarization does not change on propagation through the material. However, in linear dichroism, the polarization can change significantly. An extreme example of this effect occurs if the dichroism is strong enough that each grain of a polycrystal acts like a polarizer. As is well known from light optics, a pair of crossed polarizers will block the beam, but a third one oriented at an intermediate angle to the other two and interposed between them will increase the transmission. This effect is due to the rotation of the polarization by the middle polarizer and cannot be described by the line-integral model described above.

Therefore, we want to assess how important this effect could be in a realistic case. It would be a significant challenge to make a suitable sample, characterize its 3D orientation by some other means, then do dichroic tomography and be sure that any observed errors were due only to the polarization–rotation effect. Therefore, we performed simulations of small (2000 Å) polycrystals using optical constants derived from actual measurements. This simulation was done using a solver for Maxwell’s equations, thus minimizing approximations. The use of simulations allows the level of dichroism to be rapidly and arbitrarily varied in a way one cannot do in a real mater­ial.

We find that, depending on the strength of the dichroism and the number of angles and polarizations used, there can indeed be significant distortions due to the polarization rotation during propagation (henceforth PRP), including the apparent splitting of grains. However, with enough angles and polarizations, it is still possible to obtain an accurate reconstruction.

## 2. Methods

### 2.1. Digital phantoms

The material to be simulated was geologic aragonite at the O *K*-edge. Although the crystal is orthorhombic, we approximate it as having uniaxial anisotropy, as has been done in studies of mollusc shells in X-ray microscopy [see *e.g.* Olson & Gilbert (2013 ▸)]. The crystal structure is pseudo-trigonal and the strongly dichroic peak we use is attributed to the π* transition of the triangular carbonate anion. The X-ray absorption near-edge structure (XANES) of this material with polarization along and perpendicular to the *c* axis has been measured, and the real and imaginary parts of the refractive index calculated using *KKCalc* (Watts, 2014 ▸).

### 2.2. Simulations

The simulation was run in two dimensions using the *JCMWave* (https://jcmwave.com) finite-element simulation package. While the spatial region was 2D, the fields had three components. Thus, the phantom is represented as an infinite cylinder along the *z* direction (*x* and *y* being the dimensions of the simulation) and the polarization and incident wavevectors were allowed to have components along *z*. Similarly, the dielectric tensors were represented as 3×3 complex matrices produced by rotation of diagonal tensors according to the local crystal orientation.

It should be noted that this method of simulation assumes full-field illumination, like transmission X-ray microscopy, whereas the actual experiments that have been done with dichroic tomography typically use scanning methods such as ptychography or scanning transmission X-ray microscopy. Further, the calculations were all done in a coordinate system tied to the phantom, so the source and detector are assumed to rotate about a fixed sample as in medical computerized tomography (CT). We assume that the results are independent of imaging method, as do the reconstruction methods in the literature. For instance, a recent review of tomography (Withers *et al.*, 2021 ▸) discusses a number of methods of acquiring tomographic data and then discusses reconstruction, without specifying how the choice of imaging method affects the choice of reconstruction method. Scanning transmission X-ray tomography (laminography) in the soft X-ray energy range was done by Witte *et al.* (2020 ▸), with reconstruction methods making no reference to the use of a scanning method rather than full-field illumination.

*JCMwave* can run the simulation in a single script with multiple sources specified. This capability saves run time compared with running the calculation separately for each incident ray direction. However, all of the source waves in a single run must have the same magnitude of wavevector in the *xy* plane, so when the tilt of the incident beam relative to the cylinder was varied, separate runs had to be performed and the output data merged.

For each incident wave, the transmitted intensity just downstream of the phantom was recorded, providing a 1D array of intensities. These were stacked to create a sinogram, similar to that formed from a slice of a tomographic scan with rotation axis normal to the beam. In order to reconstruct the crystal orientation, it is necessary to tilt the incident beam with respect to the rotation axis of the phantom (always along *z*) with at least two independent tilt values (Marcus, 2022 ▸). Thus, the final sinogram was a concatenation of sinograms, one for each combination of polarization and tilt.

The effects of Fresnel diffraction (wavelength = 23 Å) are significant, as shown later in Fig. 2, and made the reconstructed images very noisy. To mitigate this effect, we Gaussian-blurred the transmission images with a kernel whose standard deviation is five pixels (82 Å) before conversion to optical density. While this procedure blurs the grain boundaries, the features came through clearly.

### 2.3. Reconstruction

Reconstructions were done using an iterative fit model, with the *Astra* (Van Aarle *et al.*, 2016 ▸) library providing the projections and back-projections needed for the forward and adjoint models. The absorption coefficient at each pixel is given by
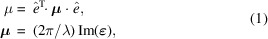
where λ is the wavelength, **ɛ** the dielectric tensor, **μ** the tensor absorption coefficient and 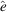 the unit vector of the polarization, in the approximation that **ɛ** is very close to 1. The quantities solved for are the six independent components of **μ**, which are described in Voigt notation as a six-component quantity (**μ**_*xx*_, **μ**_*yy*_, **μ**_*zz*_, **μ**_*yz*_, **μ**_*zx*_, **μ**_*xy*_).

The phantoms had diameters of 2000 Å and contained five randomly oriented grains in a geometry derived from a Voronoi construction (Barber *et al.*, 1996 ▸). The simulation was conducted in a 3000 Å × 3000 Å square box using plane-wave source boundary conditions. In order to change the amount by which the polarization changed on propagation, the anisotropy was modulated by introducing an anisotropy factor *a* as follows,
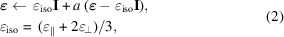
where ɛ_iso_ is the isotropic average dielectric constant, **I** the 3×3 identity matrix, and 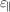 and 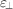 the dielectric constants along and perpendicular to the optic axis, respectively. All the dielectric constants depend on the energy of the incident X-rays. The anisotropy factor is 1 for simulating the full anisotropy and 0 for simulating an isotropic material. It was assumed that the density, and hence 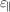 and 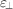, were uniform so the only variation was in the orientation, except when performing tests with an isotropic material.

Trials with isotropic grains of varying density were performed using both *Astra*’s scalar tomographic reconstruction routine (ART algorithm) and the NormalEquationsInversion routine with a Laplacian regularizer from the *Pylops* (Ravasi & Vasconcelos, 2020 ▸) library. This routine was used for all the anisotropic reconstructions. All coding was done in Python. This method is the same as was used by Marcus (2022 ▸).

The reconstructions returned a tensor at each pixel which was not guaranteed to be uniaxial. Therefore, we defined the orientation as that of the largest eigenvalue of the absorption tensor. Thus, if the reconstructed absorption tensor is biaxial and changes continuously along a path, the ‘orientation’ could change discontinuously. Similarly, the largest-eigenvalue rule makes some of the grain boundaries appear sharp despite the five-pixel blur. This orientation is displayed using a colour map in which the hue is controlled by the azimuthal orientation and the saturation by the polar angle, with an in-plane orientation indicated by a fully saturated colour and an orientation along the *z* axis by white. The orientation is a ‘double-headed arrow’; the decision of which ‘head’ to use was made by taking the one for which the *z* component was positive.

**Figure 1:**
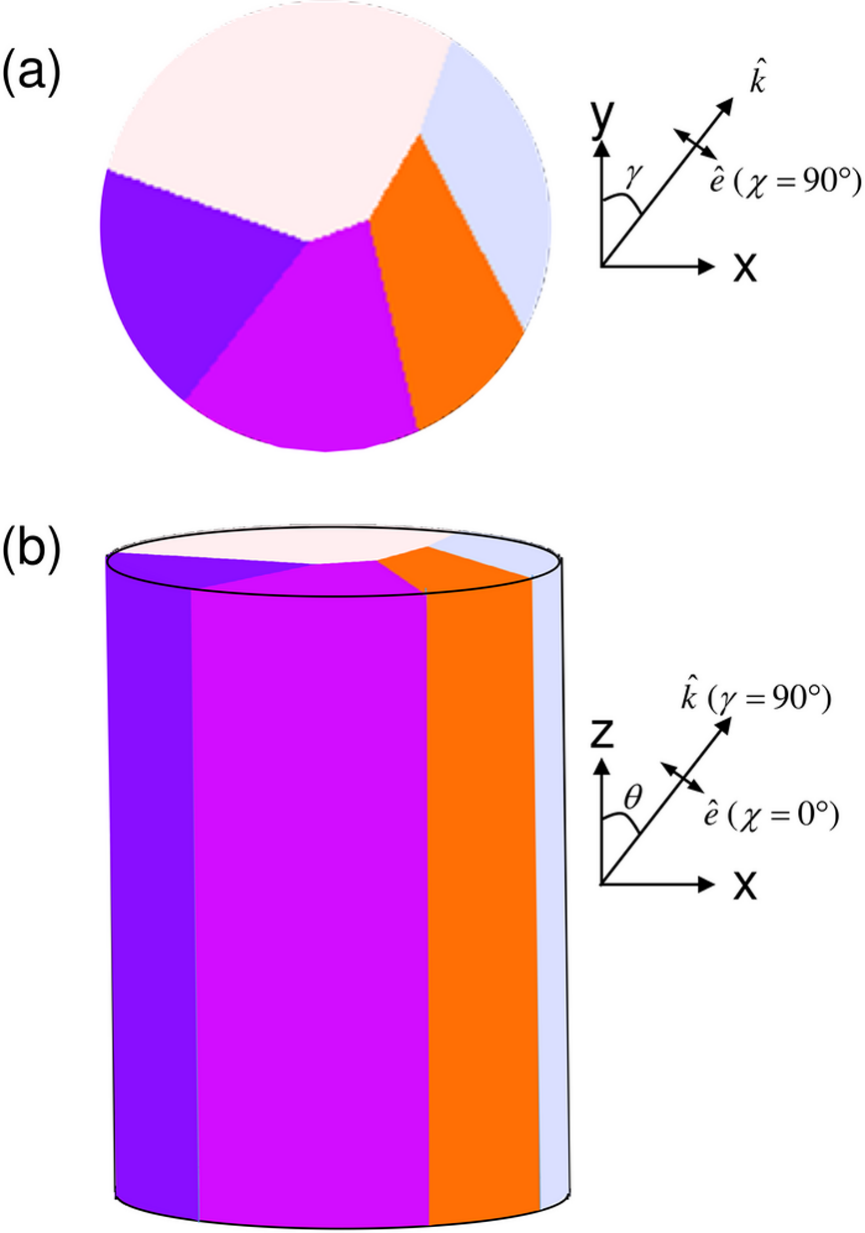
The assumed geometry, showing the coordinate system and the definition of the angles θ and γ. The incident wavevector is given by **k** = (2π/λ)(sinθ sinγ, sinθ cosγ, cosθ) and the polarization by 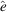 = (−cosθ sinγ, cosθ cosγ, sinθ)cosχ + (cosγ, −sinγ, 0)sinχ.

Fig. 1 ▸ shows the assumed geometry and the notation for the angles γ (azimuthal rotation), θ (tilt) and χ (polarization). The top panel shows the simulation plane and the bottom a side view of a section of the assumed infinite cylinder. Ray propagation directions 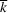 and polarization vectors 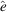 are shown.

## 3. Simulation results

Let us first consider results at an energy at which the bi­refringence is zero and the dichroism is nearly maximized, which is, with the assumed optical constants, 534.19 eV, close to the peak of absorption with polarization along the optic axis. The dielectric constants are (1 − 0.00096) + 0.0110*i* and (1 − 0.00096) + 0.00244*i* for polarization along and perpendicular to the optic axis, respectively. In all of these calculations, 72 azimuthal angles were used, evenly distributed around the circle (no missing angles). We define the polarization angle χ as 0 when the polarization is along *y* and the tilt angle so that 90° means putting the beam perpendicular to the *z* axis (CT mode).

**Figure 2:**
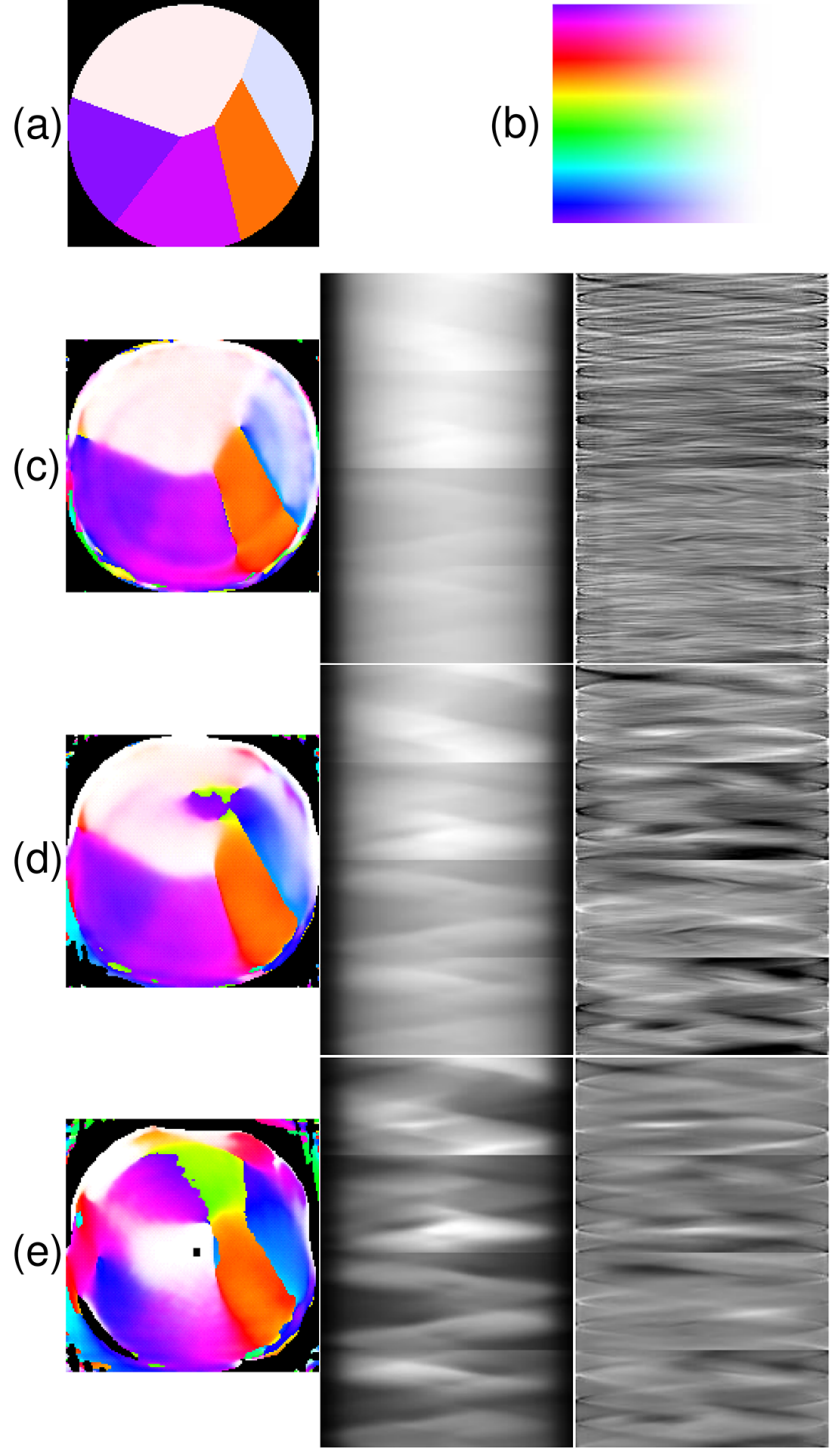
Results of the simulation and reconstruction. (*a*) The original ‘ground truth’ orientation map. (*b*) The optic-axis orientation colour map for azimuthal angles from −180° to 180° (top to bottom) and polar angles from in-plane (left) to axial (right). Panels (*c*)–(*e*) show the results of the simulation with the dichroism attenuation factor set at (*c*) 0.1, (*d*) 0.3 and (*e*) 1.0. Each panel shows the reconstruction colour map (left), the sinogram with conditions going from top to bottom (middle) and the fit residuals from the reconstruction (right). (*c*) White level of sinogram = 3.4, MSE = 2.4 × 10^−6^, black and white levels for residual = ±0.01. (*d*) White level = 3.8, MSE = 31.5 × 10^−6^, residual levels = ±0.04. (*e*) White level = 5.0, MSE = 2300 × 10^−6^, residual levels = ±0.6.

A close-to-minimal set of tilts and polarization angles (‘conditions’) is [(χ, tilt)] = [(0, 60), (45, 60), (90, 80), (126, 80)], with all angles in degrees. This set of conditions is similar to the set used by Marcus (2022 ▸). Fig. 2 ▸ shows the results, with the dichroism attenuation factors at 0.1, 0.3 and 1. In each sub-figure, the sinogram, residual (sinogram minus fit to sinogram) and reconstruction are shown, and in Fig. 2 ▸(*a*) we show the ‘ground truth’ of the phantom orientation for which the simulation was done. Note that the units on the sinogram are not arbitrary; each pixel represents the optical density for a specific position on the ‘detector’ (horizontal axis) and a specific azimuthal angle and condition (vertical axis). As expected, when the anisotropy is small, the reconstruction is accurate because the polarization changes little during beam propagation. With full anisotropy, spurious ‘grain boundaries’ and orientation gradients are introduced. This is the effect we are concerned with. That the reconstruction gets more accurate with weaker anisotropy supports the idea that we are detecting a real effect of anisotropy and not some error of modelling or reconstruction. Importantly, these erroneously detected effects may lead to erroneous conclusions on the orientational statistics or structure at grain boundaries, demonstrating the critical importance of our work. This particular realization of a random Voronoi polycrystal was not chosen to exhibit the artefact; it was the first one tried.

The simulations are conducted without considering photon noise or other error sources, so it is perhaps not surprising that the reconstruction still works well even with the anisotropy artificially reduced tenfold. Doing this in real measurements, *e.g.* by running at an energy far below or above the edge, could incur difficulties with photon statistics and measurement accuracy.

**Figure 3:**
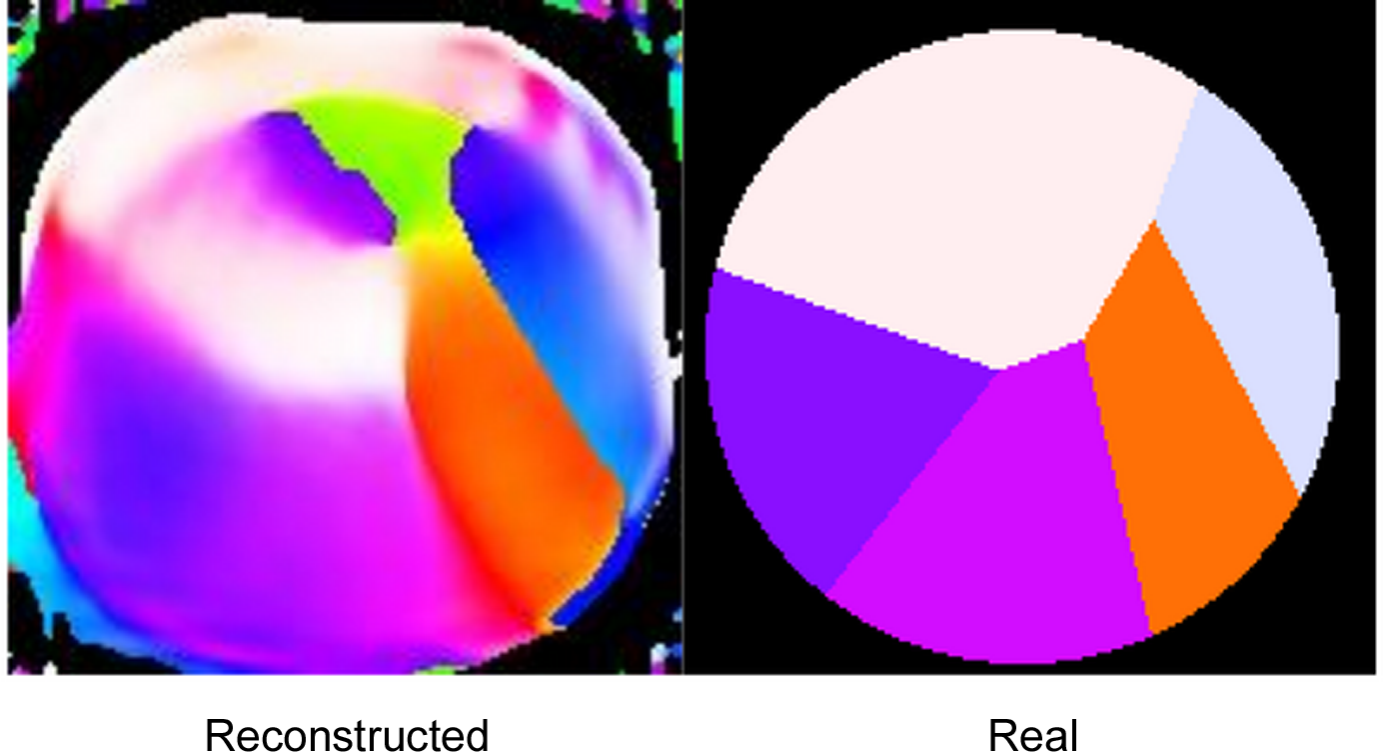
Results of simulation and reconstruction with energy set so as to maximize the birefringence, with attenuation factor 1.0.

Fig. 3 ▸ shows the result of running the same simulation at a different energy, 533.67 eV, at which the birefringence is maximized. Here, the dielectric constant is (1 + 0.00357) + 0.00582*i* with the polarization parallel to the optic axis and (1 − 0.00062) + 0.00187*i* for the perpendicular orientation. The attenuation factor is set to 1. The normalized mean-square fit error (MSE) is 0.0037, which is greater than that at maximum dichroism, 0.0023. The results are very similar to those found for the zero-birefringence energy, suggesting that the particular energy used is not critical to the effect.

**Figure 4:**
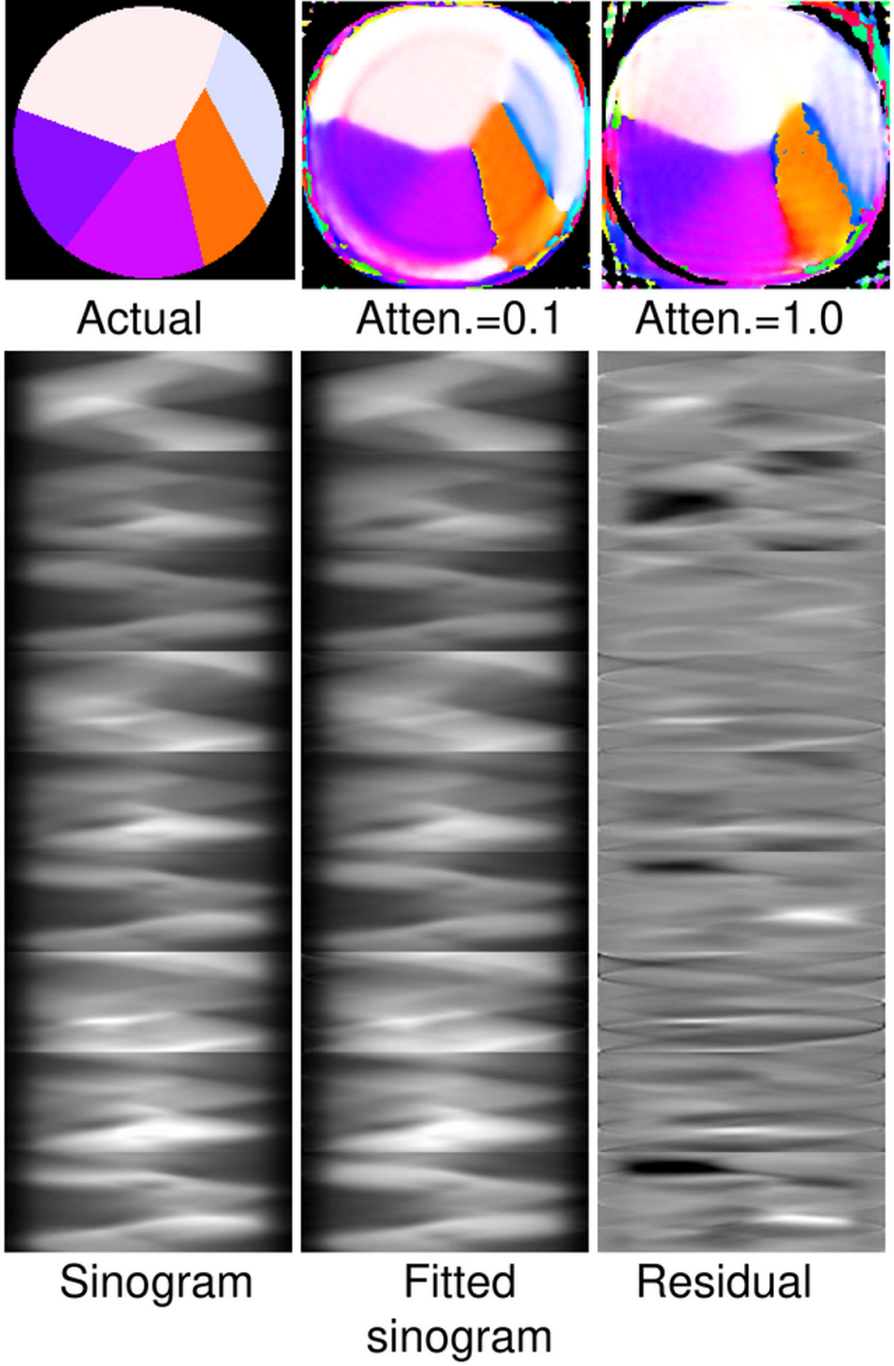
Use of a larger set of conditions for the ‘experiment’. (Top, left to right) The actual orientation map, the reconstruction with an attenuation factor of 0.1 and the reconstruction done with full anisotropy. (Bottom, left to right) The sinogram at full anisotropy, the reconstructed sinogram and the residual. The white level is 6.4, the residual levels are ±0.8 and the MSE is 0.0047.

Next, we tried a larger set of conditions, with [(χ, tilt)] = [(0, 90), (45, 90), (90, 90), (0, 60), (45, 60), (90, 60), (0, 45), (45, 45), (90, 45)] at the zero-birefringence energy. The results for attenuation factors of 0.1 and 1.0 are shown in the top panel of Fig. 4 ▸, and the sinogram, fitted sinogram and residual for full anisotropy in the bottom part. Now the reconstruction looks good even for full anisotropy, even though the fit is not very good, with an MSE of 0.0047. Compared with the case shown in Fig. 2 ▸, the residual appears to have a relatively small fraction of pixels showing a poor fit (black or white). We surmise that the reason the reconstruction works is that the redundancy provided by takng measurements at many conditions allows the reconstruction to be dominated by sinogram pixels in which the errors caused by PRP are relatively small. In other words, the ‘extra’ conditions provide consistency checks.

### 3.1. One-dimensional propagation tests

**Figure 5:**
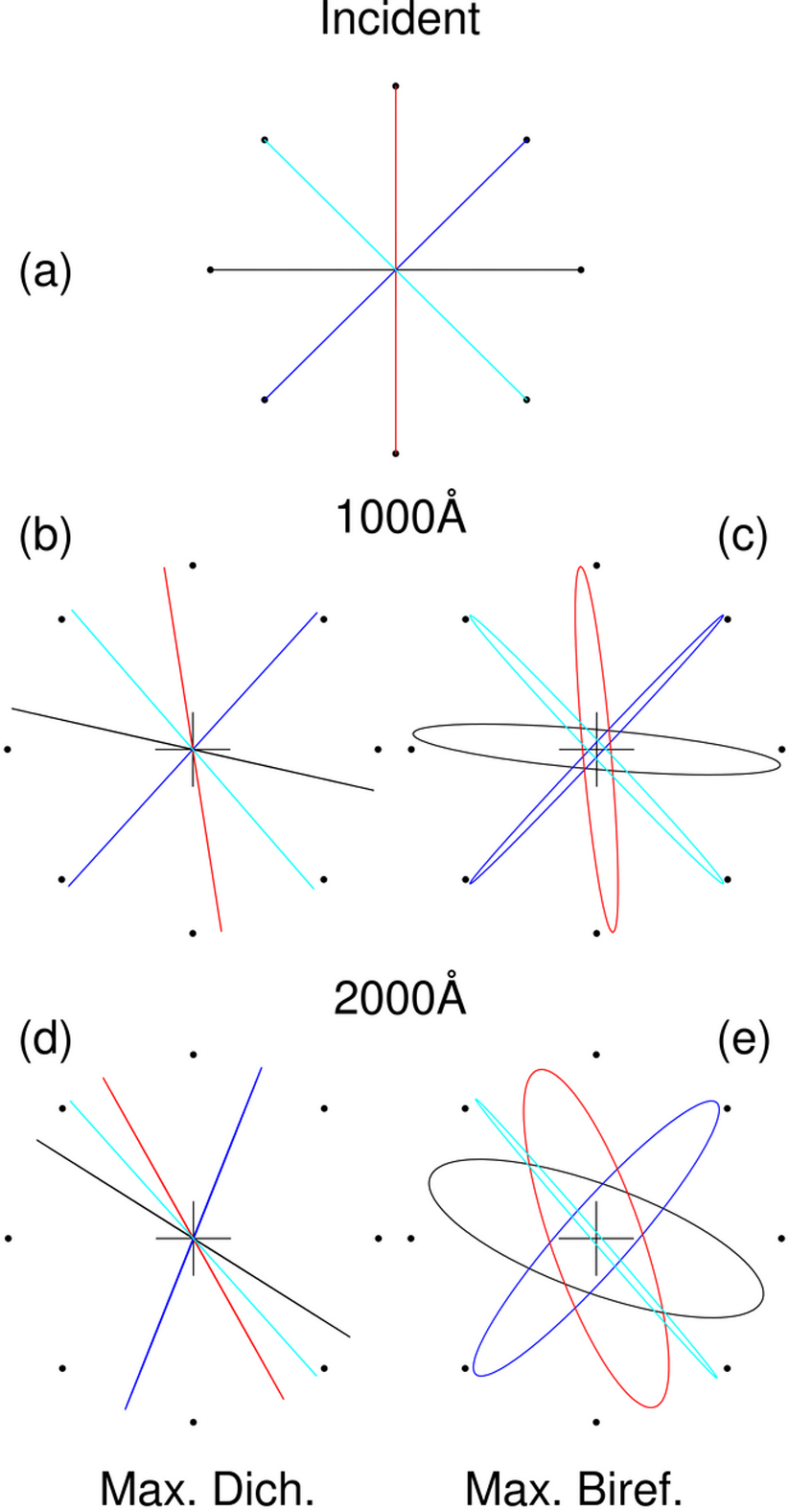
Evolution of polarization propagated through a 1D model. The trace of the polarization vector, normalized to constant magnitude, is shown in each panel. The circle of dots in each panel shows the incident orientation. The colour coding indicates which incident polarization goes with each plotted transmitted polarization. (*a*) The incident polarization. (*b*)–(*e*) The polarization after propagation through 1000 Å is shown in (*b*) (maximum dichroism) and (*c*) (maximum birefringence), and the polarization after propagation through 2000 Å is shown in (*d*) (maximum dichroism) and (*e*) (maximum birefringence).

As a way of getting an idea of how the polarization changes as the beam propagates through the sample, we implemented a 1D (multislice) model using the approximation shown in equation (7) of the report by Chang *et al.* (2020 ▸). This approximation neglects scattering and reflection at interfaces between areas with differing optical constants. We divide a 2000 Å path length into 200 slices, with the orientation either remaining the same as the previous slice’s or changing to a new random orientation with a probability per slice given by
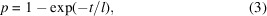
where *t* is the slice thickness and *l* the persistence length, which we keep at 500 Å. The orientation of the first slice is random. We then start a ray with a given polarization and propagate it through the sample, recording the polarization at the end. The calculation was done with the optical constants listed above for maximum dichroism and for maximum bi­refringence, with four incident polarizations. Fig. 5 ▸ shows the polarizations after propagating through 0 Å, 1000 Å and 2000 Å of the ‘sample’. At maximum dichroism, where the birefringence is small, the polarization remains linear but rotated, while at maximum birefringence the polarization becomes elliptical. We see that the polarization rotations can become significant, in agreement with the Maxwell solver simulations.

## 4. Diagnostics

How would an experimenter know that there is a significant error due to the PRP effect? We can use the above results as a guide to what to look for. We have seen already that the fit obtained during reconstruction degrades as the strength of the dichroism increases. As shown in Fig. 2 ▸, the fit becomes poor for certain areas and angles.

If the sample is everywhere optically uniaxial, we can also use the biaxiality of the reconstructed absorption tensor as an indicator. A uniaxial material will have, for each voxel, an absorption tensor **μ** with two equal eigenvalues. Thus, we can create a rotationally invariant normalized measure of bi­axial­ity as follows.

**Figure 6:**
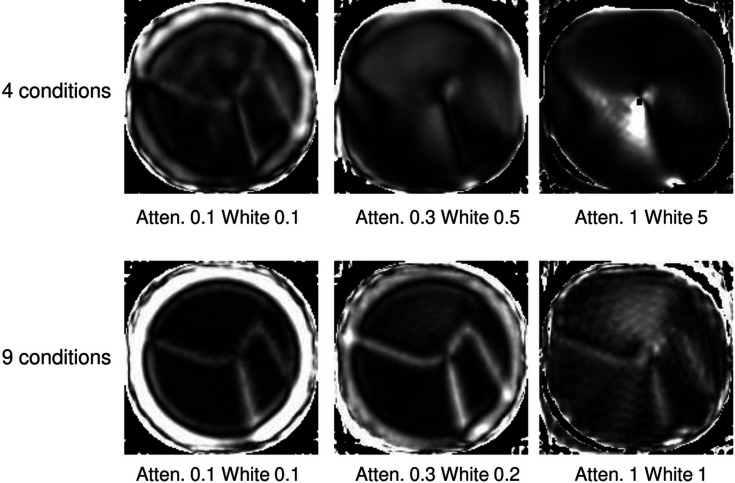
Biaxiality plotted for the reconstructions shown in Fig. 2 ▸ (top) and Fig. 4 ▸ (bottom). The dichroism attenuation levels and the white level for the biaxiality are shown below each image.

Assuming the material has greater absorption along the optic axis than perpendicular to it, we sort the eigenvalues of the absorption tensor for each voxel into ascending order: *e*_0_ ≤ *e*_1_ ≤ *e*_2_. If the material had greater absorption in the perpendicular directions than parallel, then we would simply reverse the sorting order. We define the isotropic part as *e*_iso_ = 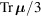 = (*e*_0_ + *e*_1_ + *e*_2_)/3. The biaxiality measure is then
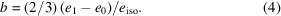
This measure is plotted out for the cases above in Fig. 6 ▸. We see that the biaxiality is large where the reconstruction fails to replicate the actual orientations of the ‘sample’. For the favourable cases, the biaxiality is small and mostly confined to the grain boundaries. These are areas in which each resolution element can contain material of multiple orientations, so it ‘reads’ as biaxial. For the areas in which the reconstruction does not match the actual ‘sample’, the biaxiality measure is greater than 1. These areas include the ‘split’ grain and fuzzy white area in Fig. 2 ▸(*c*).

If a redundant set of angular conditions were used, the data could be reconstructed with a subset of these conditions. Having the fit error or biaxiality increase when using subsets would be an indication that redundancy helped reduce the PRP error.

It should be noted that biaxiality could appear as a result of the actual sample structure, even if the material is nominally uniaxial. For instance, deformation on a scale smaller than the spatial resolution could cause the material to appear to be biaxial. However, there is a limit to how big the apparent biaxiality could be. Similarly, a defect such as a poorly crystalline or highly strained region could ‘read’ as biaxial. However, such areas would also have reduced dichroism due to the mixing of orientations in each resolution element, which would show up in the reconstruction. Of course, reduced dichroism would also appear where the material is not a pure phase, *e.g.* it contains precipitates. All of the above makes a good argument for a type of reconstruction which yields the full 3×3 absorption tensor, rather than constraining it to a uniaxial tensor with a fixed magnitude of dichroism as done by Chang *et al.* (2020 ▸).

## 5. Mitigation

We have seen that the change in polarization of the incident beam on propagation through the sample can cause significant errors in the reconstruction of orientations by dichroic tomography. How can we mitigate this effect? We have already seen one method, mainly taking more data than would be necessary just to reconstruct the orientations. The ‘extra’ data provide redundancy, which allows the reconstruction algorithm to ‘ignore’ angles at which the PRP effects are most significant. However, it may not be practical to take much more than a minimal set of data. Therefore, another approach is needed.

Far above the absorption edge, the dichroism becomes weak, so that the polarization does not change much on propagation. Therefore, the reconstruction, in the absence of noise, can be accurate. Fig. 2 ▸(*a*) shows that reducing the anisotropy tenfold essentially removes the problem. A similar effect may be obtained by confining measurements to small samples. However, this approach makes the measurement more sensitive to noise and artefacts.

### 5.1. Iterative forward modelling

Another possibility is iterative reconstruction. A straightforward approach is a fixed-point iteration scheme. We start by assuming the polarization everywhere in the sample to be the same as in the incident beam for each condition and we do the reconstruction, deriving an estimate for the absorption tensor **μ**^0^(**r**) at each voxel. Next, we simulate the propagation of light through the sample at each angle and illumination condition to derive an estimate for the polarization at each voxel, and use that as input for the reconstruction, yielding a new estimate for the absorption tensor **μ**^*k*^(**r**) in the *k*th iteration. This results in a new estimate for the orientation. This is repeated until it converges, as determined using a suitable norm such as |**μ**^*k*^ − **μ**^*k*−1^|. The routines required to make this scheme work include:

*Initialization.* Alignment, centring and setting of the polarization for each voxel and illumination condition relative to the sample.

*Reconstruction.* Given an assumed polarization for each voxel and illumination condition, and the measured sinograms, the absorption tensor is estimated. If ptychography is used, the reconstructed tensor is complex, so includes the birefringence.

*Estimation of birefringence.* Unless ptychography or another coherence-based method is used, the experimental data do not give the birefringence at each voxel. This can be estimated if one makes assumptions about the material, such as that it is uniform with known optical constants, so there is a known relation between the absorption tensor and the phase shift on propagation. This assumption is often good for biominerals and crystals of a single substance as used by Apseros *et al.* (2024 ▸). Another approach is to run at an energy at which the dichroism is large and the birefringence small. The birefringence may then be taken as zero. To do this would require taking a good XANES spectrum of the material and then applying the Kramers–Kronig transform as we did for aragonite, but using the same beamline and beam conditions as for the tomographic measurements.

*Propagation.* Running a full Maxwell simulation through the sample for each illumination condition and iteration may require an impractical amount of computation. A multislice approximation, such as was used in the 1D propagation tests, may be good enough to infer the evolution of polarization on propagation through the sample.

This iteration scheme represents a significant conceptual advance over purely experimental methods, as it directly confronts the problem. However, it is important to recognize its limitations. The approach essentially decouples the physics simulation from the reconstruction optimization, treating them as two separate problems. Furthermore, as a fixed-point scheme, this algorithm is not guaranteed to converge and can be computationally slow. However, implementing this algorithm is a natural next step.

### 5.2. Model-based iterative reconstruction framework

A more robust approach to solving the PRP reconstruction problem is to formulate it as a well posed inverse problem within the framework of model-based iterative reconstruction (MBIR).

Tomographic reconstruction is a classic inverse problem where one seeks to reconstruct the internal properties of an object from a set of external measurements (Yaqub *et al.*, 2022 ▸). This is achieved by minimizing the cost or objective, a function that typically consists of two main components: a data-fidelity term and a regularization (or prior) term. In this method, we minimize a cost function which is the sum of a term quantifying the deviation of the results of a forward model from the experimental sinograms, and a regularization term which enforces prior knowledge. In the reconstructions of Section 2, the deviation term is the MSE and the regularization term is the Laplacian. The new element in the MBIR approach is to include the propagation model in the forward model so that the polarization at each voxel is updated on each iteration. This approach contrasts with that of Section 5.1 in that there is one iterative loop, rather than two nested loops, the inner being the estimation of **μ** and the outer the propagation to determine the polarization at each voxel and condition. Because this integrated approach requires running the propagation on each iteration, it may prove to be computationally expensive even if a fast multislice approximation is used. This method is analogous to that described by Liu (2014 ▸) for dealing with polychromatic beams. In that case, what changes with propagation is not polarization but energy spectrum.

## 6. Concluding remarks

We see that tomography of materials with linear dichroism using polarized light has a feature not found in other tomographic X-ray imaging methods, namely that the fundamental assumptions of reconstruction, that the absorption through the sample may be described as a line integral of absorption coefficients for each voxel and that the found voxel values do not depend on the properties of other voxels, can be violated due to the change in polarization as the beam propagates through the sample. This effect is shown to lead to artefacts in the reconstructed orientation map.

The PRP effect may be mitigated in several ways which require taking more data (more conditions) or more precise data (operating at energies where dichroism is weak) than what would otherwise be required for good reconstruction. If certain assumptions are valid, an iterative method could be used to account for propagation effects and thus obtain a more accurate reconstruction.

## Data Availability

Data and code used for this paper may be obtained on request.
